# Narrative Development in Infant−Mother Interaction

**DOI:** 10.1111/nyas.70192

**Published:** 2026-01-20

**Authors:** Timothy McGowan, Mette Væver, Marianne Thode Krogh, Susanne Harder, Jonathan Delafield‐Butt

**Affiliations:** ^1^ Laboratory for Innovation in Autism University of Strathclyde Glasgow UK; ^2^ Strathclyde Institute for Education University of Strathclyde Glasgow UK; ^3^ Department of Psychology University of Copenhagen Copenhagen Denmark; ^4^ Centre of Excellence in Early Intervention and Family Studies University of Copenhagen Copenhagen Denmark

**Keywords:** affect, embodied social cognition, intersubjectivity, mother—infant interaction, narrative, socioemotional development

## Abstract

Narrative is a fundamental component of human cognition necessary for social meaning and cultural learning, yet its origins in preverbal infancy are not well understood. This study provides the first longitudinal analysis of the development of preverbal narrative in infancy. We measured its temporal structure in the interactions of 18 mother−infant dyads selected from a cohort of 60 dyads at 4, 7, and 10 months. Timings of infant gaze, affect, engagement duration, and progress through the four‐part narrative cycle were coded and analyzed. Interestingly, the narrative complexity of mother−infant interactions significantly increased with age; infants at 7 and 10 months reached the climax and resolution phases significantly more often than at 4 months, while also significantly decreasing in duration. Progressing through this narrative arc was strongly associated with increased positive affect, with completed narratives generating longer durations of positive affect for both infant and mother. These results identify a coherent narrative structure present in preverbal interactions that develops in complexity across the first year, strongly associated with positive feelings. This provides an affective, embodied, and participatory foundation for narrative cognition as a primary organizer of shared experience, learning, and socioemotional regulation evident from birth.

## Graphical abstract

This longitudinal study identifies a structured, four‐part narrative arc in pre‐verbal mother–infant interactions across 4, 7, and 10 months. Narrative complexity increased with infant age; older infants more frequently reached climax and resolution phases, decreasing in duration. Importantly, progressing through this narrative arc was strongly linked to enhanced positive affect for infant and mother. These findings identify a developing, affect‐rich pre‐verbal narrative structure, foundational for early cognitive and socio‐emotional development.

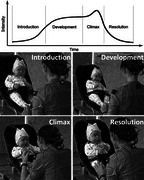

## 1 Introduction

### 1.1 Narrative

**FIGURE 1:**
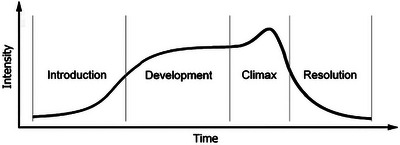
An intensity contour illustrating the four‐part narrative structure: (i) Interest in the narrative begins at a low‐intensity in the introduction, which invites participation in shared purposefulness; (ii) the coordination of the actions and interests builds during the development, with rising levels of intensity and arousal; (iii) a peak moment of excitation in mutual intention is reached at the climax; after which (iv) the intensity reduces as the purposes of the participants share a resolution, and those who were closely engaged may separate, extend, or repeat another cycle. Reproduced with permission from Trevarthen and Delafield‐Butt [15].

Narrative is a fundamental structure of human cognition and communication [1, 2]. It follows a common four‐part structure of arousal and interest that introduces a chain of events, develops over repeated cycles of action and interaction, rising to a climax of peak affect, interest, or excitement, before the tension breaks and the moment resolves into a new, quiet state of being (Figure 1). This narrative structure is common to all the time‐based arts of music, poetry, drama, and dance, and forms the foundation of literature and scientific communication. However, while narrative is often understood as a semantic sequence of statements with symbolic reference, whether vocal or gestural, the term is understood within developmental psychology to be broader.

Seminal psychologist Jerome Bruner [1, 2] viewed narrative as a foundational mode of human cognition—a way of structuring experience and making meaning through temporally organized, goal‐directed sequences of action and affect. From this perspective, narrative structure can be embodied and enacted prior to language, emerging through the temporal coordination of expressive, affective, and attentional states within interaction. Empirical analysis of the structure of early mother–infant exchanges was found to contain embodied nonverbal narrative forms that convey meaning affectively, rather than semantically [3, 4, 5, 6, 7, 8]. This has been characterized as a fundamental narrative musicality evident in the earliest interactions between infant and mother [6, 7, 8, 9, 10, 11]. This mode of enactive cognition locates the roots of narrative sense‐making in preverbal social exchange. Thus, narrative serves as a foundational mode of human cognition: a means of structuring experience and generating meaning through temporally organized, goal‐directed sequences of action and affect [4, 12, 13]. From this perspective, narrative precedes linguistic and symbolic representation and need not depend on them. Rather, a narrative can be embodied and enacted, shared through a reciprocal temporal coordination of expressive, affective, and attentional states within the interaction. In this manner, narrative is present in the earliest human interactions between parent and infant, suggesting it is a common temporal organization invariant in human social cognition from birth through to the complex cultural and linguistic intelligence of adulthood [4, 14, 15].

Narrative is structured into its four phases by distinct psychological characteristics necessary to generate its form, and within which social meaning is organized [12]. The introduction initiates an engagement with shared attention. With sustained attention to the interests, feelings, and intentions of the other, the narrative develops and builds its interest and excitement with increasing investment of time, affection, and cognitive resources. As the engagement proceeds, rising arousal and interest is expressed in the intensity of gesture and vocalizations exchanged in reciprocal acts, until a peak moment of excitation as the engagement culminates in a climax. This climax results in the release of arousal and tension before the narrative resolves, and the intensity of interest and sustained attention recedes. The participants are free to renew a shared focus or disengage. From infancy to adulthood, this pattern appears invariant [1, 2, 3, 4, 13, 14, 15]. Infants experience and embody these narratives in early interactions with caregivers and intimate others from the earliest stages of life, enabling otherwise solitary subjective experience to become intersubjective and shared [6, 10, 16, 17, 18, 19].

These interactions form “proto‐conversations” [5, 20, 21] involving reciprocal, multimodal exchanges that generate rising intensity and interest, and create shared affective meaning within a narratively structured framework [6, 18]. Initially, these structures were described as “pre‐ or proto‐narrative envelopes” [22, 23], which develop in infancy into linguistic stories in childhood, with a common framework. These early preverbal narratives establish patterns of affect, intention, and action to form a template for shared meaning on which linguistic expression can be structured [15, 16].

For many years, the dominant view in developmental psychology held that narrative engagement required language and abstract intelligence [24]. However, more recent work has shown that even newborns can cocreate narrative through movement, gesture, and vocalization—suggesting that language is not required [4, 25]. This follows insights by Malloch [6] and Trevarthen [8] into the shared rhythm and reciprocal qualities of preverbal interaction that altogether form musical narratives of shared interests, affect, and expression [10]. Comparative cognitive analysis by Dautenhahn [26] proposed preverbal narratives as foundational to human social consciousness, and seminal psychologist Jerome Bruner long recognized “narrative structure… inherent in the praxis of social interaction before it achieves linguistic expression” [1, p. 77]. Evidence from early adult–infant interactions supports the idea of narrative not merely as a product of intersubjectivity, but as an innate structure that forms an organization basis of experience in time for learning cultural practices and cooperative activity, right through to complex linguistic and technical skills of law, science, or mathematics [1, 2, 12, 18, 19, 27, 28, 29]. It is also worth noting that this narrative‐based perspective aligns with other accounts that emphasize the temporal and affective contours of lived experience. For instance, Stern's notion of vitality contours captures the dynamic shape of expressive movement [30], Rossmanith and colleagues’ concept of action arcs similarly highlights the rhythmic, goal‐related unfolding of infant actions [31], and dynamical systems models [32] describe interaction as a process of continuous coregulation between partners. Narrative complements these perspectives by offering a framework for the shared temporal organization of interactional sequences, capturing the progression from initiation, through escalation and climax, to resolution, while remaining compatible with dynamical analysis of moment‐to‐moment coordination.

### 1.2 Dyadic Interaction

Dyadic interactions are an infant's first experience of face‐to‐face dialogue. These engagements involve attention to the emotions, intentions, and arousal of another individual, creating a state of primary intersubjectivity [21, 33]. Such interactions include a level of sustained attention to the interests, feelings, and intentions of the other, but vary in duration [34]. They play a key role in regulating arousal and affect in both infant and adult, with autonomic systems in both parties becoming attuned to their social engagement systems [35]. For example, mothers modify their touch in response to infant affect, which in turn is influenced by the type of touch they receive [36]. These physiological systems support infant motivation, activity, and the learning of cultural patterns through cocreated, embodied experiences and their expected structure of arousal development, climax, and resolution [17, 37].

From birth, infants can be seen to participate in these dyadic interactions [4, 25], building experience of narrative patterns in cocreated engagements with their caregivers. As their sensorimotor system develops, their repertoire of behaviors increase to support greater complexity of attunement and coregulation, including emotional expressions, oral movements, and vocalization [21]. Increasing skills such as following gaze and prospective control of movement enhance attunement [38, 39, 40]. Vocalizations become increasingly coordinated, and the turn‐taking rhythm of adult conversation is refined [41]. These behaviors foster complex intersubjective states and support the development of more elaborate narrative structures. Around 9 months, infants begin to share attention toward external objects—forming secondary intersubjectivity [42]—and eventually, language‐based dialogue. Despite this trajectory, narrative engagement through infancy has not yet been systematically mapped.

Understanding the narrative's role in dyadic regulation has important consequences for wider adaptive child development. Poor regulatory capacities have been linked with academic performance, psychopathology, social competency, and health risk behavior in later life [43, 44, 45]. While infants can use self‐regulatory behaviors (e.g., gaze aversion [46]), they remain reliant on adult feedback [43, 47]. Dyadic regulation also supports the development of more effective self‐regulatory strategies for deployment in later childhood and adult life [47, 48, 49].

Narrative forms a fundamental architecture in human interaction and cognition [1, 2]. Its origins lie in early development, helping us interpret others’ intentions and properties of the world [4, 15]. Early narrative interactions underpin healthy sociocognitive development [50, 51], and as such, examining how these engagements unfold in infancy—and how they impact affect in the dyad—remains a key concern in developmental psychology, with relevance for infant health, early childhood education, and caregiver–child connection.

### 1.3 The Current Study

This study aims to (i) explore the temporal nature of dyadic narrative interactions and their development through the first year of life, and (ii) to examine the relationship of these temporal characteristics with the degree of positive affect experienced within an interaction. This is achieved through an analysis of mother−infant naturalistic interactions at three time points in the first year of infancy: 4, 7, and 10 months of age.

The study will help to develop a psychological understanding of narrative in infancy, expand knowledge of the link between infant affect and narrative, and form the foundation of future work to investigate how this shared social structure might be common or deviate from norms within broader consideration of social and emotional development, health, and learning.

We hypothesized that the duration of narrative engagements will increase with age as infant sensorimotor and cognitive capacities develop, enabling more sustained and complex interactions. We expected this to be true not only for overall engagement duration but also by the complexity of the narrative produced. We further hypothesized that engagements involving infants aged 7 or 10 months will more likely reach the climax or resolution phases of the narrative cycle, while a greater proportion of engagements were expected to terminate in the introduction and development phase in early infancy. With regard to positive affect, we expected this to be a function of narrative completion, with an increase in positive affect where narrative cycles are completed as opposed to those that simply initiated and developed, but did not reach a climax or conclusion.

## 2 Materials and Methods

### 2.1 Participants

Mother−infant dyadic interaction data analyzed in this study were collected at the University of Copenhagen Babylab between 2007 and 2013, and held within the Centre of Excellence in Early Intervention and Family Studies at the University of Copenhagen. Mother−infant pairs were recorded longitudinally as part of a wider project to examine interpersonal dynamics in mother−infant interaction and their relations to development and health from birth to 13 months of age. The original study dataset included both healthy mothers and those diagnosed with postnatal depression; the data examined for this study comprised only of mother−infant dyads where the mother was considered healthy.

Healthy first‐time mothers were recruited to the project during pregnancy, with the following exclusion criteria applied: premature birth of the infant, nonsingleton pregnancy, mental or physical disabilities in the infant following birth, drug or alcohol abuse in the mother, psychopathology in the mother, the development of a severe neurological condition in the mother in the first year following birth, and the development of a severe somatic health condition in the mother in the first year following birth. Mother and infant could not be included in the cohort if they lived too far from the testing location, or if prebirth interviews (to administer the Present State Examination [52], as well as assessments of attachment status and personality pathology) were not completed. The study was approved by the Ethical Review Board at the University of Copenhagen, and all mothers gave written informed consent prior to participation.

**FIGURE 2:**
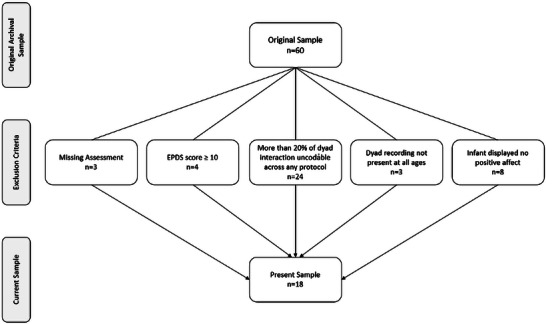
Flow diagram to show the selection of participants in the present study. This figure shows a flow diagram to illustrate the selection of the sample used in this study based on its inclusion and exclusion criteria required for this analysis.

Recruitment resulted in a sample of 60 typical mothers and their infants. Further, for this study, we applied additional exclusion criteria (fully summarized in Figure 2) based on the usability of audio‐video recordings and availability of auxiliary data to create the final sample for analysis, as well as an EPDS score equal to or above 10 administered 6–8 weeks postpartum. This was to ensure the integrity of the video footage analyzed, and that the included engagements were a true representation of mother−infant interactions and not overly impacted by missing data. The result was a longitudinal cohort of 18 mother−infant dyads. The mean age of mothers from these dyads was 31.2 (range 23−43), with eight of the infants being boys. All mothers were White Europeans, spoke Danish as their first language, and had a mean level of education of 15.7 years, including primary school (range 12−16 years).

### 2.2 Procedure

**FIGURE 3:**
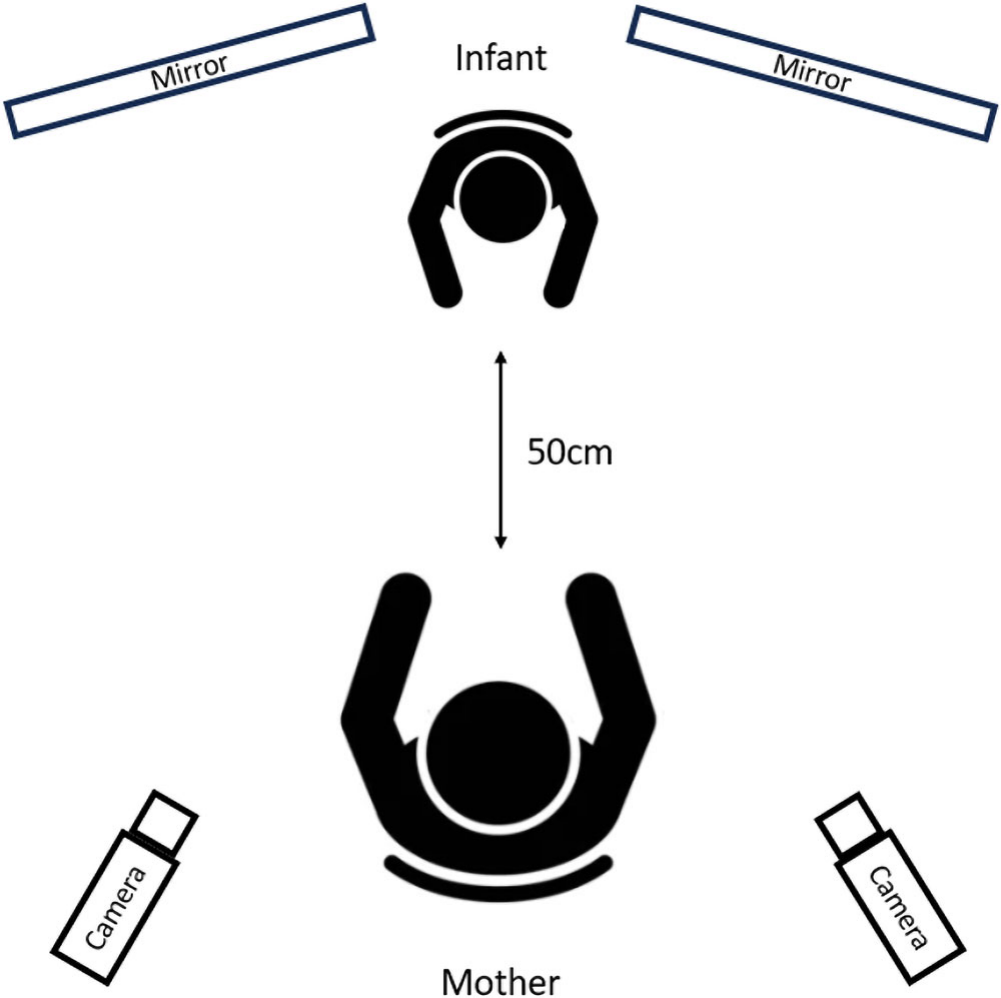
Experimental setup for mother–infant interaction. This figure illustrates the manner in which mother and infant began their period of engagement. Mother and infant were initially seated 50 cm apart, with recording equipment set up behind the left and right shoulders of the mother. Mirrors were placed behind the left and right shoulders of the infant so as to enable the recording equipment to capture a frontal view of the mother during interaction. Mothers were instructed to behave with their infants as they normally would.

Mother−infant dyads attended an observation room at the University of Copenhagen Babylab when the infants were aged 4, 7, and 10 months. The Bayley Scales of Infant and Toddler Development—Third Edition [53] were completed for each infant at all three time points, and visits were timed with the infant's eating and sleeping routines to ensure they were in an alert state. Mother and infant sat opposite one another in a standard face‐to‐face setup with the infant in a baby seat and the mother on a chair [54], beginning their interaction approximately 50 cm apart (Figure 3 illustrates the precise nature of the experimental setup). Mothers were instructed to interact with their infants in a natural fashion as they normally would, but no toys were provided, and mothers were requested to avoid using a pacifier during the engagement. The interaction was filmed using a Panasonic NV‐GS300 from two perspectives at a rate of 25 frames per second. The precise setup of these two perspectives varied between two possible options. Option one utilized one camera to record a frontal view of the infant from behind the mother's shoulder and a second camera to capture a lateral view of the dyad. Option two used one camera positioned behind the mother's left shoulder (facing the infant) and a second camera positioned behind her right shoulder (also facing the infant). For a dyad to be included in this study, at least one recording from behind the mother needed to be in the data archive (dyads with only lateral recordings were excluded and were counted as recording not present). Further to this, two mirrors placed behind the infant allowed cameras to capture a frontal view of the mother (dyads where the mother's or infant's faces were obscured for more than 20% of the recording were excluded—see Figure 2). The interaction lasted 10 min in total, but was terminated early if the infant cried for more than 30 s continuously or if the mother felt the infant to be too unsettled.

#### 2.2.1 Narrative Coding

Narrative phase (introduction, development, climax, or resolution) was coded on a second‐by‐second basis across the opening 5‐min segment of recorded interaction, using Behavioral Observation Research Interactive (BORIS) software [55], and temporally aligned with the infant gaze and affect data (see below). The coding protocol for narrative structure was developed by Delafield‐Butt et al. [51], based on nonverbal narrative paradigms in neonates [4, 10] and children [27]. The procedure first identified periods of active engagement between mother and infant. For this study, engagement was defined as beginning when infant gaze was coded as “gaze on” for ≥2 s, and terminating when infant gaze was “gaze off” for ≥2 s. Gaze was used to delineate engagement, as visual attention to a dyadic partner's face indicates readiness for interaction and underpins face‐to‐face encounters [56]. Both mothers and infants are attuned to each other's gaze direction, with infant head movements and visual tracking approaching adult levels by 3–4 months of age [57]. Moreover, gaze control and head tilting allow infants to achieve “subtle instant‐by‐instant regulation of contact” [58, p. 502]. A minimum gaze duration of 2 s was chosen to exclude fleeting or glancing looks, while remaining inclusive of gaze toward other features (e.g., the partner's hands) during the interaction.

Infant gaze was prioritized in the analysis, as mothers were instructed to remain engaged with their infants, resulting in “gaze on” being present for ≥92% of frames in pilot coding. In addition, mother gaze in the wider video catalogue could not be coded with the same accuracy due to the rear camera placement (making it difficult to ascertain whether the mother's gaze was directed at the infant or near the infant). As such, interactions were defined as periods of adult–infant engagement initiated by a minimum of 2 s of infant gaze toward the mother and ending when gaze was broken for more than 2 s (unless the infant remained visibly engaged by, for example, looking at their mother's hands).

**FIGURE 4:**
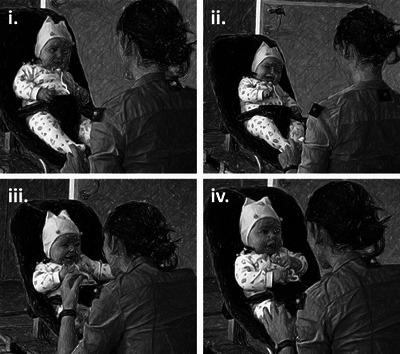
A narrative cycle between mother and infant (aged 4 months): The interaction begins with an (i) **introduction** phase, when shared attention is established between members of the dyad and a multimodal reciprocal exchange is initiated. In some cases, this exchange begins to enter a (ii) **development** phase with a build in energy and intensity over repeated reciprocal cycles of action and interaction shared between the two, which may culminate in a (iii) **climax** phase of peak energy and intensity often accompanied by vocalization and heightened positive affect. Following this peak, the energy and intensity of the interaction may subside into a (iv) **resolution** phase before the interaction comes to a close.

Each engagement was then labeled according to the most advanced narrative phase reached. For example, if the interaction followed a narrative trajectory and peaked in energy and intensity without proceeding to joint resolution, it was classified as “climax.” This labeling was informed by the intensity and arousal observed during the engagement (see Figure 4 for exemplars of each narrative phase). Specifically, and building on the Delafield‐Butt et al. [51] protocol, (i) *Introduction* was coded when an initiating action from one dyadic partner—interpretable as communicative—was acknowledged and reciprocated by the other. A reciprocal act was understood to be any change in expression (e.g., a smile), action (e.g., kick of a leg or a flick of an arm), or vocalization. This phase could involve several reciprocal actions, provided there was no clear escalation of arousal or intensity. (ii) *Development* was coded when arousal and intensity increased, expressed through heightened movement, vocalization, or facial–bodily expression. This could involve growing volume or pitch (acoustically), or increasing velocity or acceleration (kinematically). Each developmental phase was, therefore, marked by an observable and temporally sustained rise in expressive intensity, rather than by purely subjective impression. This approach diverged slightly from Delafield‐Butt et al. [51], who defined development based solely on reciprocal response without requiring increased arousal. (iii) *Climax* was coded when a clear peak of energetic intensity was reached following the development phase, across any expressive modality (acoustic, kinematic, or facial–affective). This was typically evident in the most pronounced movement, vocalization, or expressive outburst before a subsequent reduction in arousal. (iv) *Resolution* was coded when a sustained reduction in arousal followed a climax, and a mutual calm was re‐established between partners. Coders were trained to identify these parameters through repeated viewing of exemplar sequences and discussion of boundary cases to establish consistent application of the coding scheme, and periodically reviewed their coding for reliability across sessions. In keeping with the narrative model, each coded phase assumed the presence of all preceding phases (e.g., climax must be preceded by introduction and development). Periods of uncodable gaze lasting more than 2 s were not considered breaks in engagement unless accompanied by other indicators of disengagement (e.g., infant turning away, or maternal disengagement). As with gaze and affect, 20% of recordings were double‐coded, and inter‐rater reliability was calculated (time‐based κ = 0.71; event‐based κ = 0.68) [59]. Time‐based κ was used to assess agreement across all time points in the coded sequence, providing a measure of how consistently coders aligned in identifying behaviors moment‐by‐moment across continuous time. Event‐based κ was used to ascertain agreement in identifying the occurrence and type of discrete behavioral events, regardless of their precise temporal alignment. Together, these two measures provide complementary assessments of reliability.

#### 2.2.2 Gaze Coding

Gaze coding identified periods of engagement and disengagement within which affect and narrative phases could subsequently be coded. Gaze was coded on a frame‐by‐frame basis over the first 5 min of each interaction using the video annotation software ELAN [60]. Gaze was coded into three categories: gaze on, gaze off, and not codable. For gaze on to be coded, the infant needed to have visually attended to the mother's facial area for a minimum period of two frames (80 ms). If this criterion was not met, then gaze off was recorded (this approach was adapted from Beebe et al. [61]). The third criterion, not codable, was used where infant gaze could not be ascertained (e.g., if the infant's face was blocked from view by the mother). In such cases, the code immediately preceding the not codable period was recorded. If during more than 20% of an interaction, it was not possible to ascertain infant gaze, the dyad was excluded from analysis. Blinking was not coded as gaze off, but if an infant's eyes remained closed for more than 280 ms, gaze off was recorded. Twenty percent of dyadic engagement was dual‐coded, and inter‐rater reliability was calculated (time‐based κ = 0.83, event‐based κ = 0.69) [59].

#### 2.2.3 Affect Coding

Infant and mother affect was coded on a second‐by‐second basis to assess affective displays over the course of 5 min of interaction. Infant affect was classified according to six categories: high positive, low positive, neutral interest, mild negative, high negative, and uncodable. The protocol was an adapted criterion developed by Høskuldson and Smith‐Nielsen [62] based on a similar procedure used by Koulomzin et al. [63] and Beebe et al. [56]. BORIS was used to annotate video recordings. Recordings where the infant displayed high levels of fussiness, minimal engagement with the mother, or no positive affect were removed so as to mitigate outlier impact. Finally, 20% of recordings were dual‐coded, and inter‐rater reliability was calculated (time‐based κ = 0.79, event‐based κ = 0.81) [59]. For the purposes of our analysis, the two positive affect categories were collapsed into one.

#### 2.2.4 Analysis and Generalized Linear and Linear Mixed Models

We were interested in the extent to which the temporal structure of an engagement developed through infancy (with regard to patterns of arousal, in intensity, and the precise form of narrative structure) as measured at 4, 7, and 10 months of age, and whether infant affect during an engagement was related to the narrative phase reached during interaction. Generalized linear models (GLMs) and generalized linear mixed models (GLMMs) were conducted utilizing the log link (duration data, family = poisson) or logit link (binomial data, family = binomial) and the *lme4* package *glm* and *lmer* functions for GLMs and GLMMs using R [64].

Each model included a by‐participant slope for variables that varied within participant in an effort to keep random effects structures maximal where possible [65]. In cases of nonconvergence or singular fit within the maximal model, random slopes followed by random intercepts were removed until a convergent, nonsingular model was created. For example, the composite cognitive, motor, and language Bayley‐III Scale scores were included in each model, but only retained if the model successfully converged. The final structure utilized in our analysis is outlined in each section below.

## 3 Results

### 3.1 Duration

#### 3.1.1 Dyadic Engagement Durations Reduce With Age

**FIGURE 5:**
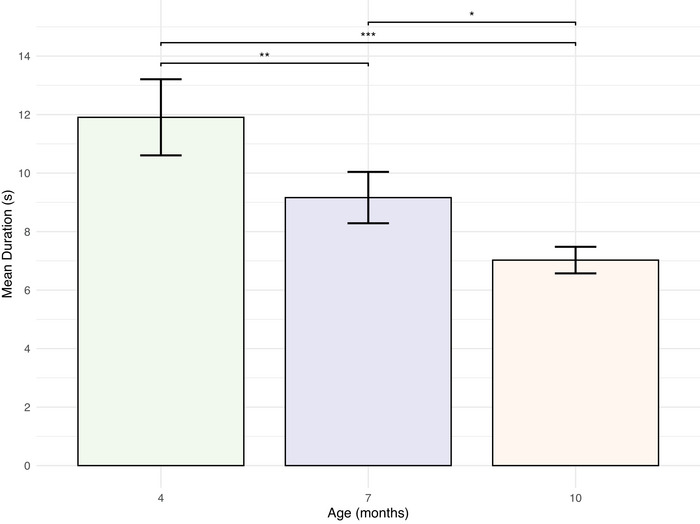
Mean engagement duration of mother−infant dyads by infant age. M**e**an duration of engagements across three infant age groups. As infants grew older, the duration of the dyadic engagements decreased. Error bars represent the standard error of the mean. *p < 0.05, **p < 0.01, ***p < 0.001.

As infants grew older, the duration of their continuous engagements decreased (Figure 5). To test whether or not engagement duration varied as a function of age, we built a linear mixed‐effects model (see Table 1 for comparisons). This model had engagement duration as our dependent variable, infant age as a fixed effect, a random effect for participant, and included a total of 467 observations. Interactions between 4‐month‐old infants and their mothers (M = 11.90 s [1.30]) were significantly longer than when the infants were 7 months (M = 9.16 s [0.88]) and 10 months of age (M = 7.03 s [0.45]). We next compared the length of engagements when infants were 7 and 10 months old (with a total of 335 observations included in the model). Infant–mother engagements were significantly longer when infants were 7 months old.

**TABLE 1:** Linear mixed‐effects model predicting engagement duration by infant age.

Comparison	β	SE (β)	*t* (df)	*p*
4 versus 7	−2.65	1.28	−2.06 (462.68)	0.004^**^
4 versus 10	−5.04	1.17	−4.33 (461.81)	<0.001^***^
7 versus 10	−2.25	0.89	−2.50 (332.16)	0.013^*^

*Note*: Comparison is between infant age groups. Engagement duration decreased significantly across age groups.

*p < 0.05, **p < 0.01, ***p < 0.001.

#### 3.1.2 Engagement Durations Reduced With Age, Despite Their Narrative Complexity

**FIGURE 6:**
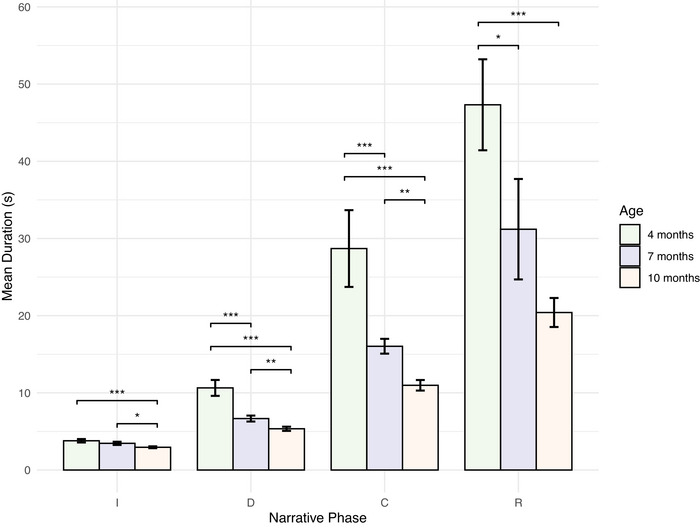
Mean engagement duration by narrative phase reached and age. M**e**an duration of engagements according to the final narrative phase the engagement reached and infant age. The duration of engagements reduced with age, regardless of their increasing narrative complexity. Error bars represent the standard error of the mean. Abbreviations: C, climax; D, development; I, introduction; R, resolution. *p < 0.05, **p < 0.01, ***p < 0.001.

We next extended this finding by considering the relationship between age and engagement duration when interactions were grouped according to the narrative phase the engagement reached (i.e., if the interactions within an engagement reached the climax phase, it would be grouped accordingly). The following results are presented in Figure 6 and reported in Table 2. We built a series of linear mixed effects models with each focusing on a different group (introduction phase group—182 observations included; development phase group—166 observations included; climax phase group—78 observations included; resolution phase group—38 observations included). Each model followed the same structure, with a dependent variable of interaction duration, a fixed effect of infant age, and a random effect of participant.

**TABLE 2:** Linear mixed‐effects models of engagement duration by age and narrative phase.

Phase	Comparison	β	SE (β)	*t* (df)	*p*
Introduction	4 versus 10	−0.81	0.24	−3.40 (178.10)	<0.001^***^
	4 versus 7	0.29	0.26	−1.10 (175.91)	0.274
	7 versus 10	0.52	0.22	2.32 (121.99)	0.022^*^
Development	4 versus 10	−5.27	0.81	−6.52 (159.52)	<0.001^***^
	4 versus 7	−3.83	0.89	−4.32 (159.57)	<0.001^***^
	7 versus 10	−1.43	0.45	−3.16 (114.41)	0.002^**^
Climax	4 versus 10	−18.69	3.16	−5.91 (73.85)	<0.001^***^
	4 versus 7	−12.93	3.41	−3.79 (73.95)	<0.001^***^
	7 versus 10	−4.80	1.36	−3.52 (10.22)	0.005^**^
Resolution	4 versus 10	−27.22	6.25	−4.35 (35.00)	<0.001^***^
	4 versus 7	−17.07	7.08	−2.41 (34.41)	0.021^*^
	7 versus 10	10.13	4.98	−2.03 (26.02)	0.052

*Note*: Comparison is between infant age groups. Engagements shortened significantly across narrative phases with infant age.

*p < 0.05, **p < 0.01, ***p < 0.001.

Interactions between 4‐month‐old infants and their mothers (M = 3.79 s [0.22]) that only reached the introduction phase were significantly longer than when the infants were 10 months of age (M = 2.95 s [0.12]), but there was no significant difference when infants were 7 months old (M = 3.46 s [0.21]). However, in the development phase (4m: M = 10.6 s [1.03]; 7m: M = 6.67 s [0.38]; 10m: M = 5.35 s [0.27]), climax phase (4m: M = 28.7 s [4.98]; 7m: M = 16 s [0.95]; 10m: M = 11 s [0.68]), and resolution phase (4m: M = 47.3 s [0.89]; 7m: M = 31.2 s [6.51]; 10m: M = 20.4 s [1.88]), both the 7 month age group and 10 month age group had significantly shorter interactions than the 4 month age group.

Each model was also subsetted to exclude engagements when infants were 4 months old, and compare the length of interactions when infants were 7 and 10 months old (introduction phase group—124 observations included; development phase group—118 observations included; climax phase group—59 observations included; resolution phase group—32 observations included). In the introduction phase, interactions were significantly longer when infants were 7 months old, a pattern repeated for the development phase and climax phase. The difference between interactions was approaching significance between age groups in the resolution phase. Altogether, these results demonstrate a pattern of decreasing engagement duration with age, despite how complex those narratives might be.

### 3.2 Narrative Phase Reached Within an Engagement

#### 3.2.1 Distribution of Completed Narrative Cycles by Age

To begin this analysis, we first wanted to determine whether or not narrative cycles that reached the resolution phase (completed narratives) were evenly distributed across all age groups. A chi‐square goodness of fit was performed, and completed narratives were not found evenly distributed across age groups (χ^2^(2) = 12.67; *p* = 0.002).

#### 3.2.2 Narrative Complexity Increases as Infants’ Age Increases

To explore this further, we investigated whether the narrative phase ultimately reached during an interaction was associated with infant age. We again designed a series of general linear mixed effect models that considered this with regard to each narrative phase separately. The “narrative phase reached” variable was dummy coded so that it consisted of two levels in each model—the “narrative phase reached” that was the focus of the model, and then all other narrative phases collapsed. Each model had “narrative phase reached” as a dependent variable, while infant age group and mean‐centered engagement duration were included as fixed effects, and participant ID as a random effect (mean‐centering is a technique where the mean of a variable is subtracted from each individual data point, resulting in a new variable with a mean of zero). Each model contained a total of 467 observations.

**FIGURE 7:**
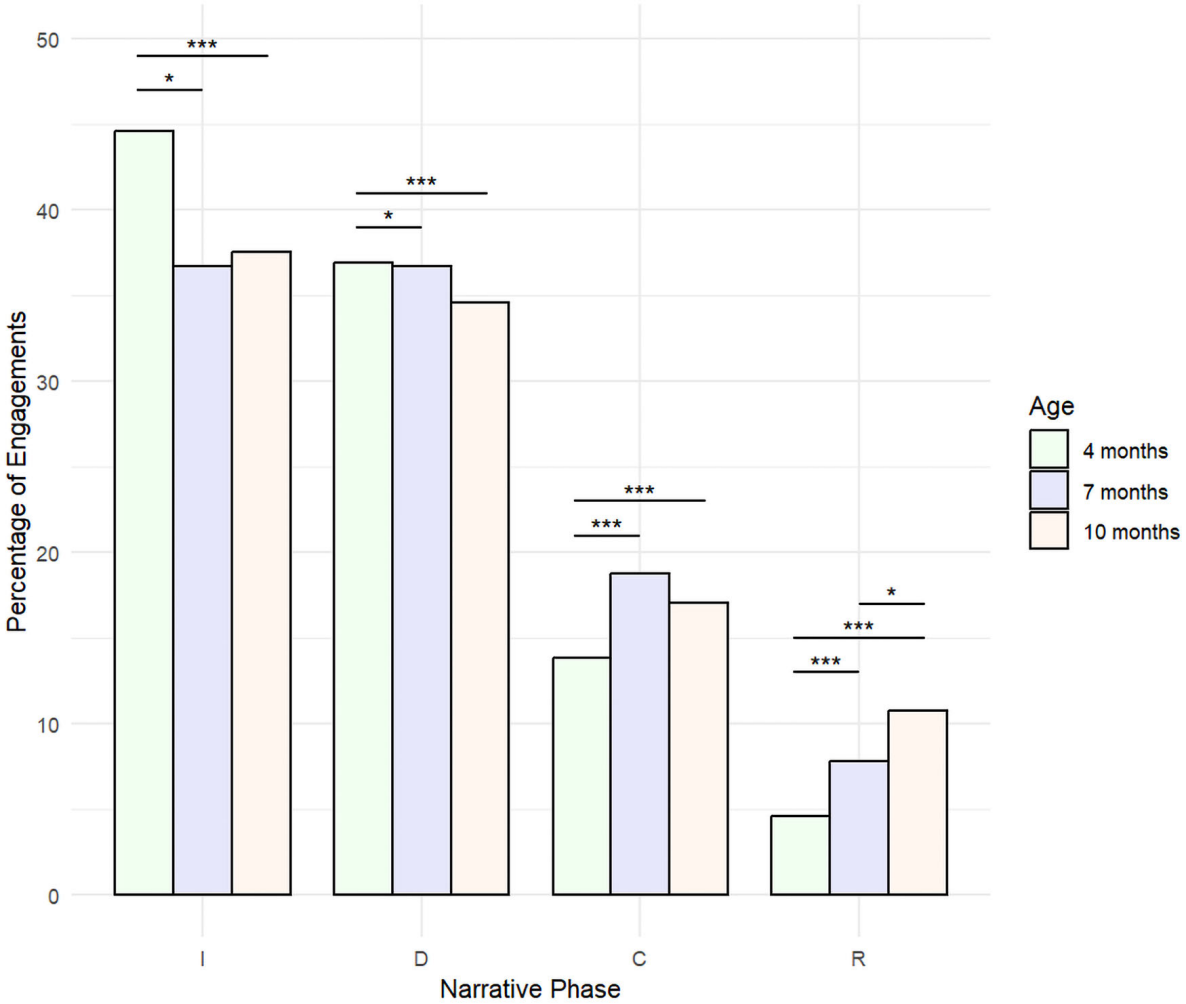
Percentage of engagements that reached a given narrative phase. The percentage of engagements based on the furthest narrative phase that was reached during a continuous interaction. The complexity of engagements increased as infants aged. Abbreviations: C, climax; D, development; I, introduction; R, resolution. *p < 0.05, ***p < 0.001.

As shown in Figure 7 and summarized in Table 3, there was a significant difference in the number of interactions that only reached the introduction phase between age groups—there were significantly more of these interactions when the infants were 4 months of age than when infants were 7 months of age, and significantly less when infants were 4 months old than when infants were 10 months. These results were reflected in the number of engagements that reached the development phase.

**TABLE 3:** Mixed‐effects models predicting narrative phase reached by infant age.

Narrative phase	Comparison	β	SE	z	*p*
Introduction	4 versus 10	−1.13	0.34	−3.56	<0.001^***^
	4 versus 7	−0.78	0.37	−2.13	0.034^*^
	7 versus 10	−0.40	0.32	−1.24	0.216
Development	4 versus 10	1.26	0.35	3.56	<0.001^***^
	4 versus 7	0.83	0.38	2.17	0.030^*^
	7 versus 10	0.48	0.33	1.43	0.152
Climax	4 versus 10	2.97	0.58	5.11	<0.001^***^
	4 versus 7	2.49	0.59	4.24	<0.001^***^
	7 versus 10	0.59	0.39	1.52	0.129
Resolution	4 versus 10	4.58	1.16	3.94	<0.001^***^
	4 versus 7	3.64	1.08	3.36	<0.001^***^
	7 versus 10	1.61	0.65	2.46	0.014^*^

*Note*: Comparison is between infant age groups. Narrative complexity increased significantly with infant age.

*p < 0.05, ***p < 0.001.

However, in the climax phase and resolution phase, there were significantly more engagements that reached these phases when infants were 7 and 10 months of age than when infants were 4 months old.

As in previous analyses, each model was then subsetted to exclude engagements when infants were 4 months old, and so compare the number of engagements that reached the introduction phase, development phase, climax phase, and resolution phase when infants were 7 and 10 months old (see Figure 7). Each model contained a total of 335 observations and is fully summarized in Table 3. Significantly more interactions reached the resolution phase for infants at 10 months of age than at 7 months of age. However, there was no significant difference between age groups concerning the number of interactions that reached the introduction, development, or climax phases.

Across all interactions, 38 engagements reached the resolution phase. These were distributed across 16 of the 18 dyads, with individual dyads contributing between 1 and 5 resolution‐phase engagements (M = 2.4). Thus, the capacity to reach full narrative resolution was not limited to a small subset of dyads but was broadly represented across the sample.

### 3.3 Narrative and Affect

#### 3.3.1 Complex Narratives Associated With Increased Duration of Positive Infant Affect

We found that infants displayed more positive affect as their engagements increased in narrative complexity, that is, narratives that came to a climax and resolution were more joyful than those that only came to a climax, which were in turn more joyful than those that just developed.

**FIGURE 8:**
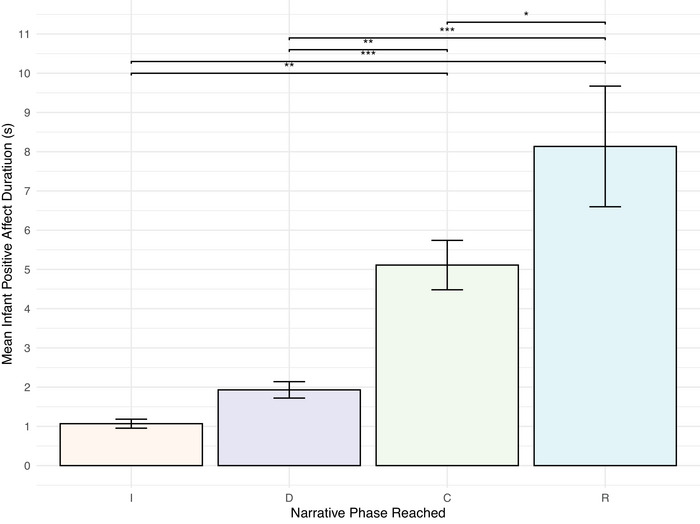
Mean duration of positive infant affect by narrative phase. Mean positive affect duration experienced by infants during an engagement, depending on the furthest narrative phase the engagement reached. Increased duration of positive infant affect was associated with more complex narratives. Error bars represent the standard error of the mean. Abbreviations: C, climax; D, development; I, introduction; R, resolution. *p < 0.05, **p < 0.01, ***p < 0.001.

To test for relations between the infants’ positive feelings and the development of narrative complexity, we calculated how durations of infant positive affect displayed during an engagement varied as a function of the furthest narrative phase reached in an interaction. We developed a linear mixed effects model with mean‐centered positive infant affect duration as our dependent variable; narrative phase reached, mean‐centered interaction duration, and mean‐centered positive mother affect duration as fixed effects; and participant, age group, Bayley‐III cognitive composite score, Bayley‐III language composite score, and Bayley‐III motor composite score as random effects. Interaction duration was included as a fixed effect to control for the impact of longer engagements upon positive affect duration. The model had a total of 442 observations. As shown in Figure 8 and summarized in Table 4, an engagement contained significantly different durations of positive infant affect depending on the furthest narrative phase reached during the interaction. There were significant differences in this regard between all narrative phases, with the exception of the introduction (M = 1.07 s [0.12]) and development (M = 1.93 s [0.21]) phases.

**TABLE 4:** Mixed‐effects model of infant positive affect duration by narrative phase reached.

Comparison	β	SE (β)	*t* (df)	*p*
Introduction versus Development	0.46	0.39	1.16 (419)	0.652
Introduction versus Climax	2.20	0.59	3.72 (428)	0.001^**^
Introduction versus Resolution	−4.24	0.89	−4.76 (423)	<0.001^***^
Development versus Climax	1.74	0.55	3.22 (423)	0.008^**^
Development versus Resolution	−3.78	0.83	−4.57 (422)	<0.001^***^
Climax versus Resolution	−2.04	0.77	−2.65 (416)	0.041^*^

*Note*: Infant positive affect increased significantly with narrative complexity.

*p < 0.05, **p < 0.01, ***p < 0.001.

Engagements that reached the climax phase (M = 5.11 [0.63]) resulted in significantly longer durations of positive infant affect than those that reached the development phase and those that only reached the introduction phase. Engagements that completed a full narrative cycle—reaching the resolution phase (M = 8.14 s [1.54])—resulted in significantly longer durations of positive infant affect than engagements that reached the climax phase, development phase, or the introduction phase.

#### 3.3.2 Complete Narrative Cycles With Resolutions Were Associated With Longer Durations of Positive Mother Affect

**FIGURE 9:**
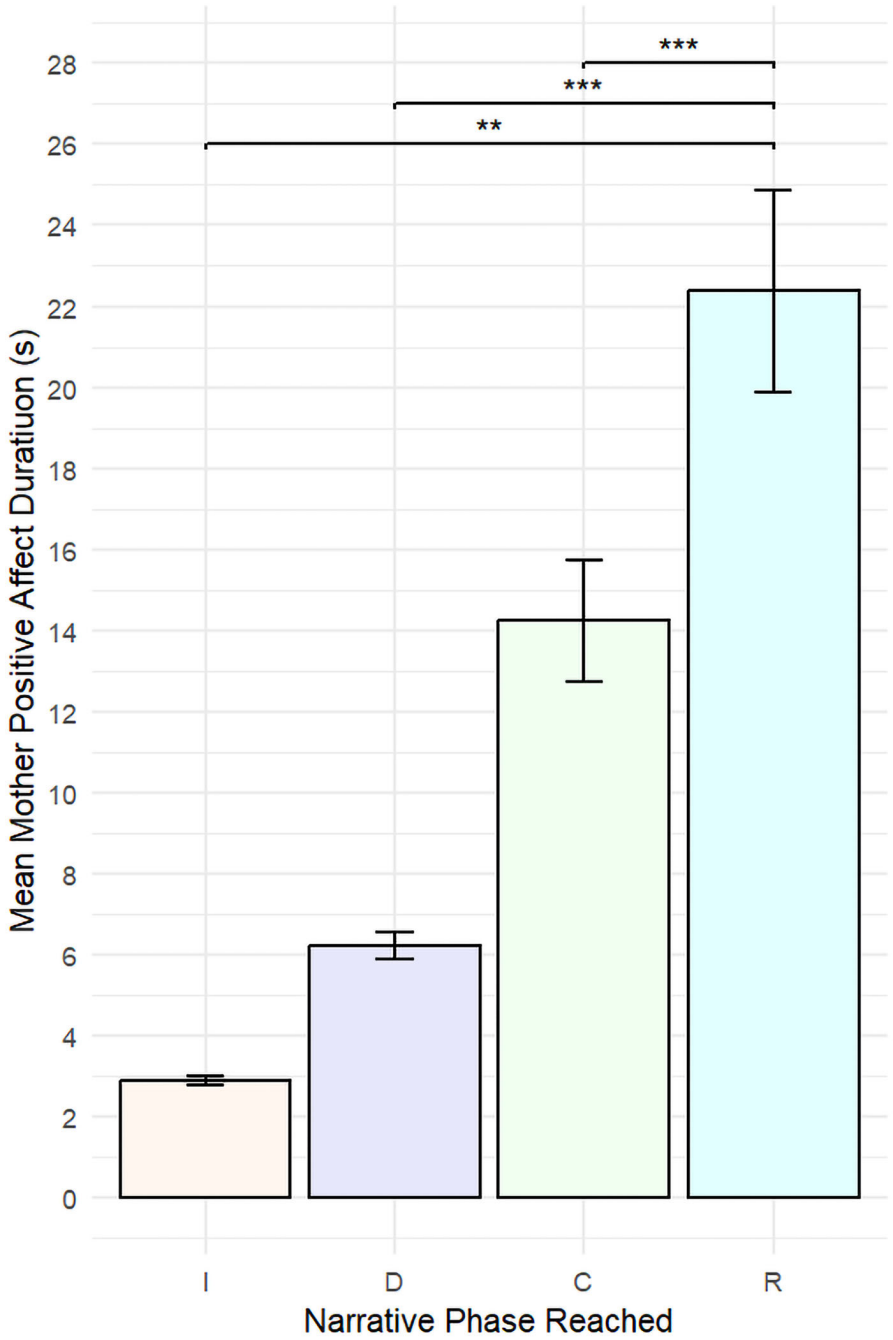
Mean duration of positive mother affect by narrative phase. Mean positive affect duration experienced by mothers during an engagement, depending on the furthest narrative phase the engagement reached. Longer durations of positive mother affect were associated with complete narrative cycles, which include a resolution phase. Error bars represent the standard error of the mean. Abbreviations: C, climax; D, development; I, introduction; R, resolution. **p < 0.01, ***p < 0.001.

Our final analysis explored the relationship between mothers’ positive affect and the furthest narrative phase reached in an interaction. We built a linear mixed effects model that mirrored the structure of the model utilized to analyze infant positive affect and the narrative phase reached. Our model had mean‐centered positive mother affect duration as our dependent variable; narrative phase reached, mean‐centered interaction duration, and mean‐centered positive infant affect duration as fixed effects; and participant, age group, Bayley‐III cognitive composite score, Bayley‐III language composite score, and Bayley‐III motor composite score as random effects. Interaction duration was again included as a fixed effect to account for the impact of longer engagements upon positive affect duration. There were 442 observations. There was no significant difference in the duration of positive mother affect (see Figure 9 and Table 5) regardless of the narrative phased reached in an interaction (introduction: M = 2.89 [0.12]; development: M = 6.22 [0.34]; climax: M = 14.24 [1.5]) except with regard to the resolution phase (M = 22.38 [2.49]), which resulted in significantly longer durations of positive mother affect.

**TABLE 5:** Mixed‐effects model of mother positive affect duration by narrative phase reached.

Comparison	β	SE (β)	*t* (df)	*p*
Introduction versus Development	−0.06	0.25	−0.24 (424)	0.995
Introduction versus Climax	0.03	0.38	0.09 (388)	0.999
Introduction versus Resolution	2.16	0.58	3.74 (316)	0.001^**^
Development versus Climax	0.09	0.35	0.26 (417)	0.994
Development versus Resolution	2.10	0.53	3.93 (350)	<0.001^***^
Climax versus Resolution	2.19	0.48	4.54 (412)	<0.001^***^

*Note*: Mothers displayed significantly longer durations of positive affect during engagements that reached a resolution phase compared to all other narrative phases.

**p < 0.01, ***p < 0.001.

## 4 Discussion

Our findings demonstrate that narrative develops across the first 10 months of life with increasing complexity, affective relevance, and decreasing durations. These data show infants increasingly develop dyadic interaction into more complete narratives comprising phases of introduction, development, climax, and resolution, alongside increasing positive affect in both infant and mother as development proceeded over the first year of life. This is the development of a fundamental structure within which social cognition is generated and organized as embodied events structured in time with characteristic patterns of interest, arousal, and shared attention. They constitute the first narratives, cocreated and shared between parents and their offspring, that become a bedrock of sustained relationship, setting patterns of arousal, interest, and affect that define their experiences, and personal knowledge of each other. These are the first cocreated experiences “in which we swim” [66], the substance of our cultural knowledge.

We found that the number of completed narrative cycles (i.e., those that reached the resolution phase) increased as infants aged, and more engagements reached the climax phase before terminating (with the exception of a plateau between 7 and 10 months). Interactions that terminated during the introduction or development phases were more common at 4 months than at 7 months, and remained relatively frequent at 10 months compared to 4 months. It is noteworthy that fully completed narrative cycles accounted for a relatively small proportion of the total interaction time. This is consistent with prior reports that infants’ attention and engagement naturally fluctuate across observation periods, even during parent‐led engagement [67]. In addition, the duration of positive infant affect was significantly greater during interactions that reached the resolution phase compared to those ending at earlier phases. Interactions terminating at the climax phase were also associated with longer infant positive affect than those ending in the introduction or development phases. For mothers, only interactions that included a full narrative cycle with a resolution phase were associated with longer durations of positive affect. As narrative phases were coded by their furthest phase reached rather than time‐stamped throughout the engagement, these findings indicate that more complete narrative interactions were associated with greater overall positive affect, rather than demonstrating affective variation across successive narrative phases.

A decrease in engagement duration with age—both in overall interaction time and when engagements were grouped by narrative phase—was somewhat unexpected, as it contrasts with our predictions based on typical cognitive and social development during the first year. For example, maturation of autonomic regulatory systems, such as heart rate variability and arousal recovery, is thought to support sustained engagement and behavioral organization [68, 69]. Likewise, improvements in attentional control, affect regulation, social interest, and emerging communicative behaviors contribute to an infant's increasing readiness for reciprocal interaction [70, 71, 72]. As these systems mature, infants typically become more able to tolerate stimulation, maintain attention, and respond contingently—capacities that would be expected to support longer, more complex interactions with caregivers.

However, a decline in interaction duration with age may also reflect developmental changes in infants’ autonomous social agency. As infants develop, they become increasingly capable of initiating, modulating, and terminating interactions based on internal states, motivational priorities, and contextual affordances [73, 74]. Rather than indicating reduced capacity for sustained engagement, shorter interactions may reflect a more efficient, selective, or self‐directed style of participation. Developmental increases in motor competence and environmental awareness can lead older infants to divide their attention across a broader range of stimuli [75], leading to briefer but more purposeful episodes of engagement. This shift toward goal‐directed exploration and multimodal engagement may reduce the time spent in any one interaction without reflecting diminished social interest or affective attunement. This finding is consistent with prior research suggesting that dyadic coordination becomes more rapid and efficient with age [76], reflecting infants’ increasing capacity for self‐regulation and communicative precision. To address the impact of a novel environment on older infants, future research could capture data in the infant's home environment using more mobile motion tracking technologies—such as markerless systems (e.g., Refs. [77] and [78]) or wireless, miniaturized wearable sensors [79]—thereby reducing the impact of laboratory novelty and unfamiliar equipment on infant behavior.

Our results suggest that the most substantial changes in the temporal architecture of narrative engagements occur between 4 and 7 months of age. These findings support the notion that humans relate to one another in a uniquely social way from early infancy through primary intersubjectivity—shared attention to the interest, feelings, and intentions of the other without intermediary objects [80, 81]. This view contrasts with shared intentionality theory, which argues that a cognitive shift toward the end of the first year—marked by the emergence of joint attention to objects and cooperative activity—represents the onset of the distinctly human form of sociality [82, 83, 84, 85]. The significant developmental changes observed during early infancy, including in narrative structure, highlight the foundational role of primary intersubjectivity—direct human–human relating—in shaping the development of social and cognitive capacities.

Our findings also underscore the close relationship between narrative structure and affect. Completed narratives were associated with increased positive affect in both infants and mothers. These interactions may offer a sense of closure and coherence, supporting emotional regulation, memory consolidation, and the learning of cultural engagement norms [4, 27, 51]. Notably, interactions that ended at a climax phase were also linked to longer durations of infant—but not maternal—positive affect. This suggests that “partial narratives” may still hold developmental value for infants. Given the infant's use of gaze aversion to regulate arousal and signal disengagement [46, 86], it is plausible that some engagements coded as climax‐endings were in fact terminated intentionally by the infant in a form of “solo‐resolution.” In such cases, the infant may disengage to manage internal state and consolidate the experience independently, rather than coregulating with the caregiver through a joint resolution.

At a more fundamental level, our results point to the presence of structured, complete narratives within the first year of life, rather than as “proto‐narrative” forms proposed in Daniel Stern's earlier theory [30]. This supports the view that narrative architecture precedes language and is not dependent on it for formation. It may also invert traditional assumptions about the relationship between language and narrative. Terrace et al. [88] argue that richly developed primary intersubjectivity—the dyadic sharing of affect and attention—is a necessary foundation for the emergence of secondary intersubjectivity and, consequently, for the acquisition of symbolic language. In line with this view, our findings suggest that the temporal and affective organization of early interactions may provide precisely this developmental substrate. Additionally, recent work by Di Liberto et al. [87] highlights the role of rhythm, and by extension, musicality, in early language acquisition. Given that narrative is a core component of communicative musicality [6, 8, 10], our findings suggest that narrative structure may underlie not only early intersubjective engagement but also the development of language itself (cf. Refs. [88] and [89]). It is tempting to speculate further on its evolutionary antecedents [90].

Gallagher [91] and Sparaci and Gallagher [92] challenge this interpretation, arguing that narrative requires representational and reflective capacity, and, therefore, cannot fully emerge until later in development. In contrast, we conceptualize narrative as a foundational cognitive framework, shaping early action and cocreation of meaning. The structured, affective exchanges observed in mother−infant interactions—as early as 3 months in our data, but also in the neonatal period [4, 25] and in prematurely born infants during kangaroo care [6, 8]—demonstrate discernible narrative cycles. These findings provide compelling evidence against the notion of infant “prenarrative” ability and instead position narrative as a central organizing structure of social cognition from early development onward.

While the distinction between proto‐narrative and full narrative capacity may seem academic, it carries important real‐world implications for how we understand and support the learning of preverbal children. These implications extend beyond infant care and development into areas such as disability support and additional or special needs education. Practitioners working with children who have not yet developed linguistic skills often recognize movement and behavior as meaningful communicative expressions [93]. Understanding narrative as the underlying structure of these expressions offers parents and educators a framework for structured engagement [11, 94] that supports children in regulating internal states [19] and facilitates early learning [93, 95]. In contrast, by denigrating these engagements as “less than” their linguistic adult counterpart, the importance of those interactions can be dismissed and not attended to meaningfully. The importance of embodied narrative extends beyond infancy into formal education. Narrative enables learners to interpret experiences, understand intentions, and evaluate actions [96, 97]. Children approach learning tasks by segmenting them into manageable episodes with clear beginnings, middles, and ends—creating structured projects of sense‐making [98]. Successfully completing these narrative episodes fosters confidence, builds memory of task completion, and strengthens emotional and intentional coherence [94, 98]. Our findings suggest that the foundational structure of this narrative learning is already in place in infancy.

Additionally, while the present study focused on Western, face‐to‐face mother–infant interactions, this configuration represents only one among many cultural and ecological contexts in which early communication unfolds. In numerous caregiving settings, infants are carried, surrounded by multiple caregivers, or engaged in a variety of contexts. The narrative framework proposed here, emphasizing temporal organization, contingency, and affective arcs, can extend to these alternative modalities to account for cultural variations. For example, Gratier [17] found that Parisian mother−infant pairs used the pulse of the shared narrative differently if they were of Indian extraction, or native to France. Such cultural variation on narrative form will provide useful insight into invariant and culturally specific aspects of narrative meaning‐making.

A potential limitation of this work is that narrative phases were identified by human raters, who may be predisposed to perceive narrative structure due to the inherent narrative nature of human cognition. Human coders might project narrative organization onto interactions that do not objectively possess it. If valid, this critique could call into question the reliability of human‐coded narrative identification in infancy. However, it does not preclude the existence of infant narrative. If humans are indeed narrative beings by nature, it would follow that narrative structures emerge early in development—reflecting an innate architecture rather than a learned or culturally imposed framework.

To address this concern, we propose that future studies develop automated approaches to narrative structure identification in infant interactions. An unsupervised machine learning model, such as a neural network trained without human‐coded ground truth, could be used to assess for narrative phases independently. For example, this method could organize audio and motion data into temporally overlapping “tiles,” each representing a defined time window of multimodal data, to identify clusters based on learned patterns of similarity. Such clustering ought to reveal any underlying temporal architectures independent of human assessment to offer a complementary, computational method of analysis. Another alternative is that future work could extend our approach by modeling fluctuations in intensity over time, to examine whether modalities such as infant and maternal affective expression, arousal, or proximity follow dynamic contours aligned with the unfolding of narrative structure—potentially integrating multiple measures to fully map the complex multimodal organization of shared meaning.

## 5 Conclusion

This study presents the first longitudinal analysis of the temporal structure of narrative engagement across the first year of life and their association with infant and maternal affect. Our findings suggest that narrative architecture in infant–adult interaction is a foundational feature of early social cognition that develops rapidly in the first year and is important for positive maternal and infant affect. These results contribute to the first longitudinal mapping of nonverbal narrative in infant–mother interaction, and contribute to a growing body of evidence supporting the existence and significance of preverbal, embodied, and cocreated narrative structures in infancy—structures that scaffold attention, learning, and the development of intersubjective understanding.

## Author Contributions

T.M.G. designed and implemented the present study, conducted the data analysis, and authored the manuscript. S.H., M.V., and J.D.‐B. conceived the present study, and S.H. and M.V. conceived the longitudinal mother−infant paradigm. S.H., M.V., and M.T.K. carried out the primary data collection at the University of Copenhagen, on which the present study is based. S.H., M.V., and M.T.K. also contributed to manuscript preparation and editing. J.D.‐B. designed and implemented the present study design, supervised data analysis, and authored the manuscript.

## Conflicts of Interest

There are no competing interests to declare.

## Data Availability

The data necessary to reproduce the analyses presented here are not publicly accessible. The analytic code necessary to reproduce the analyses presented in this paper will be made available via the Open Science Framework (OSF) upon publication. The coding manuals used for video analysis will be made available via the Open Science Framework (OSF) upon publication.
